# Rapid qualitative analysis of recruitment obstacles in the FORVAD (Posterior Cervical Foraminotomy surgery versus Anterior Cervical Discectomy surgery in the treatment of cervical brachialgia) randomised, controlled trial

**DOI:** 10.1186/s13063-024-08391-4

**Published:** 2024-08-17

**Authors:** Rebecca Talbot, Ruchi Higham, Julie Croft, Gemma Ainsworth, Sarah Brown, Rachel Kelly, Deborah Stocken, Simon Thomson, Nikki Rousseau

**Affiliations:** 1 Clinical Trials Research Unit, Leeds Institute of Clinical Trials Research, University of Leeds, Leeds, UK; 2 Department of Neurosurgery, Leeds Teaching Hospitals NHS Trust, Leeds, UK

**Keywords:** Qualitative, Process evaluation, Rapid qualitative analysis, Interview, Experience, Randomised controlled trial, Surgery, Neurosurgery, Equipoise

## Abstract

**Background:**

The number of surgical trials is increasing but such trials can be complex to deliver and pose specific challenges. A multi-centre, Phase III, RCT comparing Posterior Cervical Foraminotomy versus Anterior Cervical Discectomy and Fusion in the Treatment of Cervical Brachialgia (FORVAD Trial) was unable to recruit to target. A rapid qualitative study was conducted during trial closedown to understand the experiences of healthcare professionals who participated in the FORVAD Trial, with the aim of informing future research in this area.

**Methods:**

Semi-structured interviews were conducted with 18 healthcare professionals who had participated in the FORVAD Trial. Interviews explored participants’ experiences of the FORVAD trial. A rapid qualitative analysis was conducted, informed by Normalisation Process Theory.

**Results:**

Four main themes were generated in the data analysis: (1) individual vs. community equipoise; (2) trial set-up and delivery; (3) identifying and approaching patients; and (4) timing of randomisation. The objectives of the FORVAD trial made sense to participants and they supported the idea that there was clinical or collective equipoise regarding the two FORVAD interventions; however, many surgeons had treatment preferences and lacked individual equipoise. The site which had most recruitment success had adopted a more structured process for identification and recruitment of patients, whereas other sites that adopted more “ad hoc” screening strategies struggled to identify patients. Randomisation on the day of surgery caused both medico-legal and practical concerns at some sites.

**Conclusions:**

Organisation and implementation of a surgical trial in neurosurgery is complex and presents many challenges. Sites often reported low recruitment and discussed the logistical issues of conducting a complex surgical RCT. Future trials in neurosurgery may need to offer more flexibility and time during set-up to maximise opportunities for larger recruitment numbers. Rapid qualitative analysis informed by Normalisation Process Theory was able to quickly identify key issues with trial implementation so rapid qualitative analysis may be a useful approach for teams conducting qualitative research in trials.

**Trial registration:**

ISRCTN, ISRCTN reference: 10,133,661. Registered 23rd November 2018.

**Supplementary Information:**

The online version contains supplementary material available at 10.1186/s13063-024-08391-4.

## Background

### Cervical brachialgia

Cervical brachialgia (pain, usually down the arm, caused by a trapped nerve in the neck) is common in adults aged 40–60 years old, with over 110,000 new cases reported in the UK each year. In addition, over 15% of patients with brachialgia are incapacitated due to pain, which increases the likelihood of financial hardship and reduced quality of life for the patient [1]. For the majority of patients, symptoms of brachialgia decrease over time with appropriate management (analgesia and physiotherapy).

Surgical intervention is offered for patients who experience persistent symptoms 6 weeks after onset and diagnosis of brachialgia. Studies have shown that surgical intervention can aid faster recovery over time than appropriate non-surgical management [2, 3]. However, the preferred method of surgical procedure remains a matter of clinical equipoise [4]. Two types of surgery are commonly used in the UK National Health Service (NHS) to treat cervical brachialgia. Anterior cervical discectomy (ACD), which accesses the trapped nerve from the front of the neck, with removal of the whole disc, is effective but there is a high possibility of long-term issues such as dysphagia (difficulty swallowing) (9.5%), speech problems (3.1%), and repeat surgery at other neck sites (25% risk in 10 years) [5, 6].

An alternative to ACD is posterior cervical foraminotomy (PCF), where the trapped nerve is accessed from the back of the neck. Only 25% of surgeons perform PCF and the decision to perform PCF is normally attributed to surgeon skill and preference when considering the risks of performing PCF, which can include neck pain, blood loss, and corrective surgery [7]. Emerging evidence suggests that PCF may be a more effective procedure than ACD, with better clinical outcomes and fewer long-term complications post-surgery [8, 9] but further high-quality randomised comparisons are needed to inform treatment decision making.

### The FORVAD trial

**Fig. 1:**
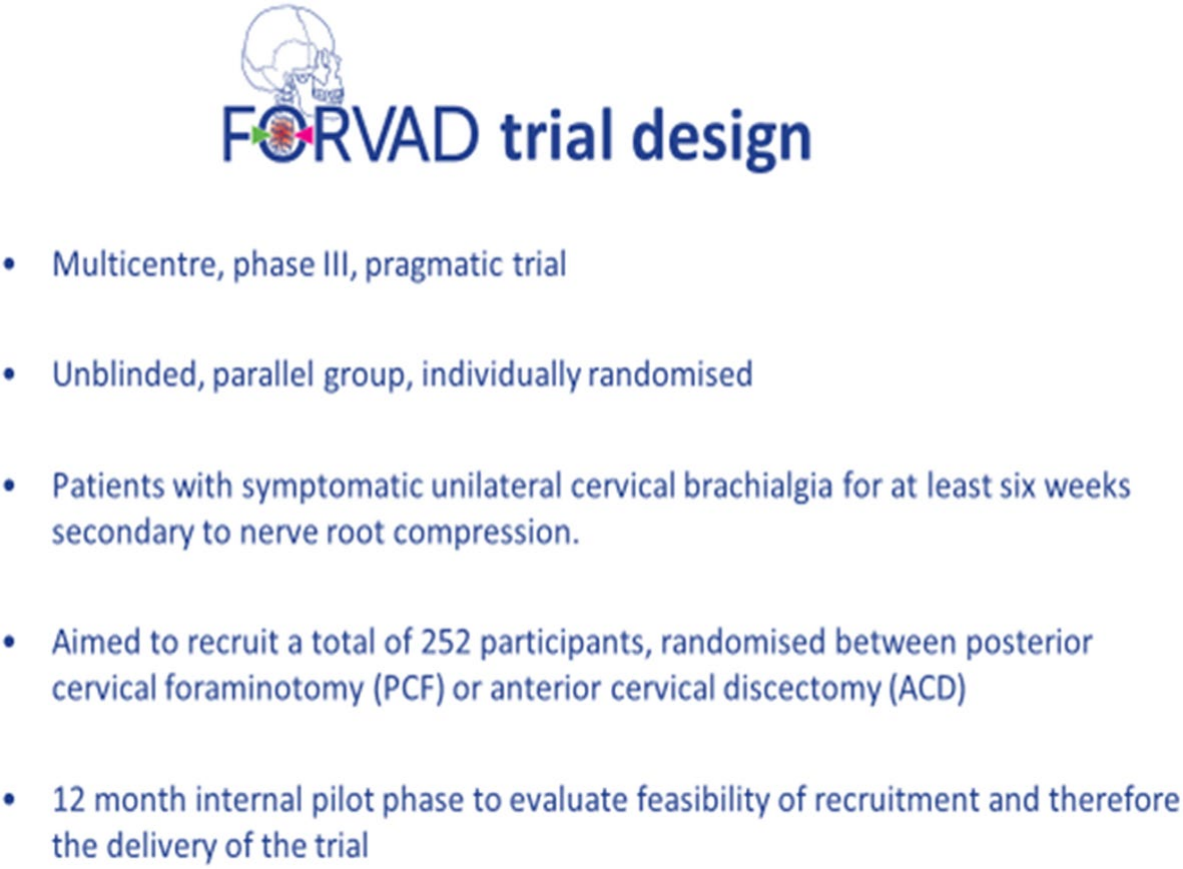
Key features of FORVAD Trial design

**Fig. 2:**
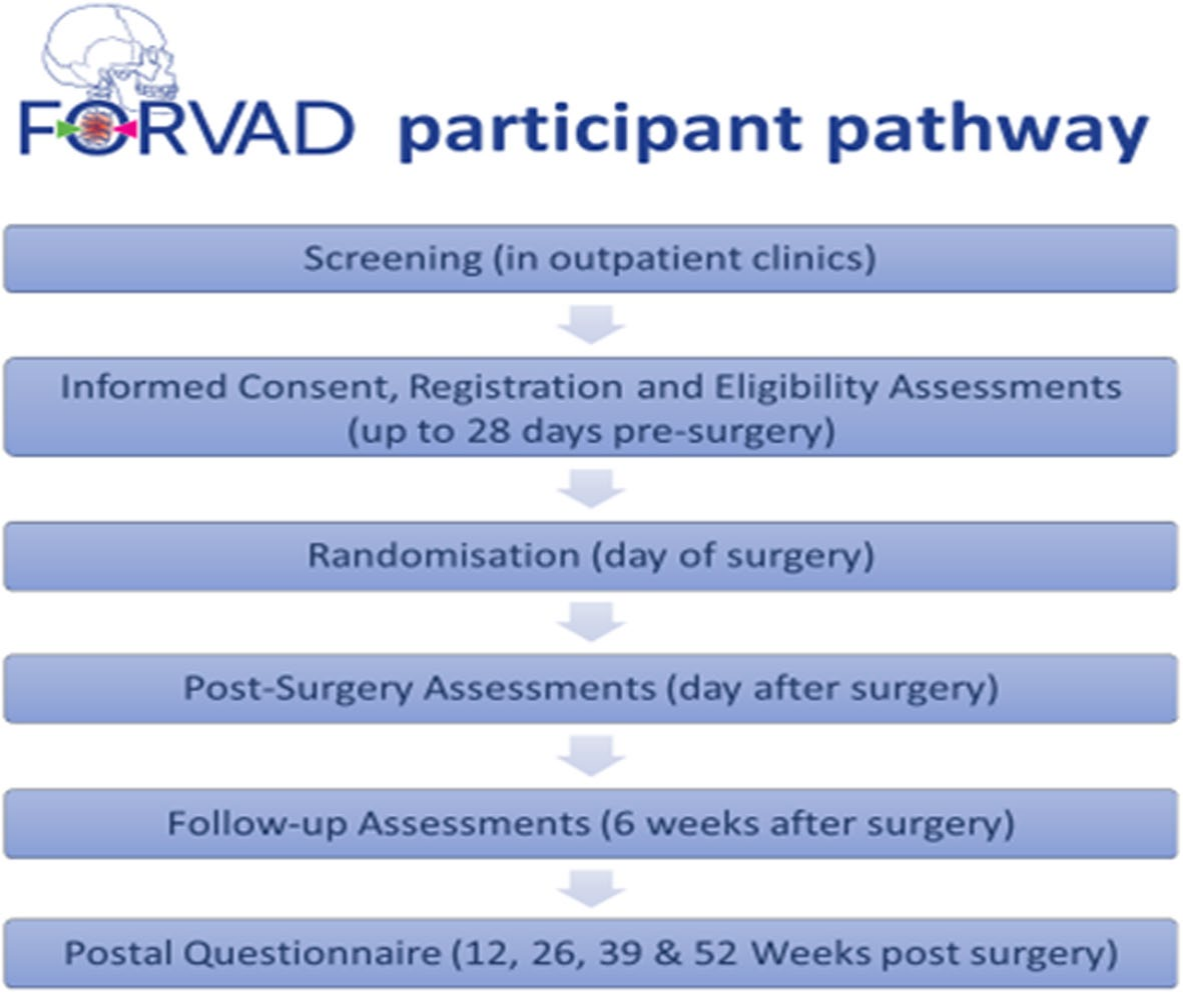
FORVAD participant pathway

The FORVAD trial (Clinical and cost-effectiveness of Posterior Cervical Foraminotomy surgery versus Anterior Cervical Discectomy surgery in the treatment of cervical brachialgia) was funded by the UK National Institute of Health Research (HTA Award Ref: 16/31/53) to address uncertainty about the most surgically effective and cost-effective management of cervical brachialgia. It aimed to recruit 252 patients from 15 hospitals across the UK, randomising them on a 1:1 basis to ACD or PCF, with a 12-month internal pilot to assess feasibility. The full trial design has been published previously [10, 11]; key features of the trial design are shown in Fig. 1 and an overview of the participant pathway is shown in Fig. 2.

FORVAD opened to recruitment in January 2019, and in the first 12 months 27 participants were registered. Twenty-three participants were randomised (against a target of 25 for the internal pilot), with four participants registered but not randomised before trial closedown. Eighteen participants were recruited at the lead site, and although 11 sites had opened, only six had registered a participant. Average recruitment during the pilot was 0.2 participants per site per month, which was below the minimum progression criteria set for the pilot (0.7). Moreover, the onset of the COVID-19 pandemic posed additional challenges for all trials. The recruitment figures attained before the pandemic indicated that reaching the target sample size for FORVAD would be difficult. Consequently, the decision was made in May 2020 to close the FORVAD trial early, with recruitment formally ceasing in June 2020.

A qualitative process evaluation had not been included in the original plans for FORVAD, making it difficult to pinpoint which factors had made recruitment for this trial particularly challenging. Whilst the trial team had informal information about the challenges the trial had encountered, it was agreed that it would be useful to collect more systematic information about the barriers to trial implementation from across participating sites during trial closedown, to inform future research.

### Qualitative research and clinical trials

Qualitative research in trials can improve understanding of trial implementation (including recruitment, participant retention, and trial set up) and of the implementation of trial interventions (including how they are experienced and interact with context). Qualitative research has been less utilised in trials involving surgical intervention [12] despite recognition that surgical interventions are complex in nature, and that qualitative research can be valuable in this context [13, 14]. Where qualitative approaches have been used in surgical RCTs [15–18], they have contributed to a greater understanding of the obstacles to successful surgical trial implementation [18–22] [17, 19, 23].

Recruitment into surgical RCTs can be challenging [24–27], [28]. Limited numbers of patients may be eligible for recruitment, especially in trials of a complex surgical nature [29]. Additionally, the number of potential patients identified as eligible for a trial can vary from site to site, based on surgeon preference alone [30]. Delays in resourcing designated trial staff may hinder trial set up, impacting the time available to recruit participants [28, 31]. Key factors influencing patient participation in trials include trust in the trial and positive communication between patient and healthcare professionals [15, 32], weighing up the benefits of taking part over the risk of surgical intervention [33], and being able to help further research in surgical areas which have been previously overlooked [16]. The initial contact is important when participating in surgical trials, as is a clear understanding of what to expect if participating in a surgical RCT [34]. Other identified issues affecting pragmatic RCTs in surgery are a lack of understanding amongst surgeons about aspects of study design, such as the value of randomisation in minimising selection bias, and aspects of surgical culture and training that are not favourable when conducting an RCT [35].

In recent years, there has been an increase in the role of surgeons recruiting patients for RCTs; yet this presented significant challenges for surgeons when conveying equipoise during the recruitment phase [36]. The concept of clinical equipoise relates to uncertainty within the expert medical community and is distinct from individual or theoretical equipoise; within clinical equipoise it is accepted that individual investigators may have preferences [37] but they are “urged to put aside personal opinions and accept the collective uncertainty of their peers in order to recruit to a trial” (4, p2). Where preferences exist, however, they may be reflected in the way that surgeons communicate information about the trial and may affect trial recruitment [4, 30, 36].

The way that trials are organised and implemented at sites impacts trial success. For example, studies have shown the role of the research nurse to be a crucial aspect of successful trial recruitment [38]; logistical issues can trial recruitment difficult [30] and the timing of recruitment in a patient’s treatment pathway can be crucial [4]. Trial organisation and implementation is often affected by site heterogeneity and facilities [19, 39].

At present, there are a paucity of studies which look at surgical trials in neurosurgery specifically [40, 41]. In addition, to the best of our knowledge, no studies have considered healthcare professionals’ experiences of surgical trials in cervical brachialgia. The clinical outcomes of the FORVAD trial have been published previously [10]. This paper presents the results of interviews conducted with site staff as part of a rapid qualitative evaluation conducted during closedown of the trial. The aim of this study was to understand why recruitment had been so challenging, with a view to informing the design of future trials in cervical brachialgia and neurosurgery.

## Methods and design

### Objectives

The objectives of the FORVAD qualitative study were to (1) understand the reasons why the trial was unable to recruit to target; (2) identify aspects of trial design and conduct that could be improved in a future RCT of surgical interventions to address cervical brachialgia and (3) identify and understand any wider issues that may need to be addressed in future randomised controlled trials in neurosurgery.

### Study timing and setting

Recruitment, data collection, and analysis took place between March and June 2021, during the closedown of the FORVAD trial.

All sites involved in FORVAD were approached to take part in the qualitative study. This was 17 sites in total, including:

1. Sites which opened to recruitment and had registered at least one participant (*n* = 6).
2. Sites which opened to recruitment but had not registered a participant (*n* = 5).
3. Sites which were in set-up but had not opened to recruitment (*n* = 6).

### Sampling, recruitment, and consent

Potential participants were identified from Authorised Personnel (Delegation) Logs for sites that were willing to take part in the qualitative sub study. All staff identified as working on the trial were invited to participate in an interview. Initial email invitations were sent out to each member of staff along with a copy of the qualitative sub-study participant information sheet (PIS). A follow-up email was sent after 3–5 days if there had not been a response from the initial email invitation. Across sites we aimed to obtain a range of perspectives by including Principal Investigators (PIs), other participating surgeons who were not the PI, research nurses and trial assistants/administrators. We aimed to interview at least one surgeon and one research nurse or non-medical professional involved in the trial at each site. All staff who responded to the email invitation were offered an interview at a time and date that was convenient to them. A copy of the verbal consent checklist was sent by email prior to the interview. At the start of the interview, participants were given an opportunity to ask any questions about the study and then verbal consent was then taken and audio recorded.

### Interview procedure

Semi-structured interviews were conducted by members of the research team (RH, RT) remotely via Microsoft Teams, with interviews lasting between 12 and 55 min. The interviews were semi-structured using a topic guide (Additional file 1) that was informed by: (1) existing experience and knowledge of qualitative research in trials within the team; (2) a sensitising framework (Normalisation Process Theory [42]) and (3) the contextual knowledge and experience of the FORVAD Trial Management Group. In summary, the interviews covered overall views on FORVAD, experiences of cervical brachialgia, surgical approach, setting up the FORVAD trial, experiences of FORVAD recruitment and experiences of delivering FORVAD trial interventions. Questions were modified where appropriate for each participant. The interviewer made notes during the interview and interviews were also audio recorded and transcribed verbatim by an approved transcription service (meeting data management requirements for transfer and storage).

### Data analysis

Analysis was undertaken using rapid qualitative analysis (RQA), an approach which has been used to enable rapid evaluation of interventions (e.g. clinical and health service models) to inform policy and practice [43]. The use of RQA allowed the researchers to undertake data collection and analysis together within a short timeframe, whilst maintaining rigour and allowing for findings to be developed iteratively throughout the data collection period. Data analysis took place concurrently with data collection. The qualitative team met twice a week throughout the data collection period to discuss the developing analysis and make decisions about further data collection. Interviewers shared their reflections of recent interviews so that similarities and differences across the interviews could be identified and categories for the analysis could be established. After the first three interviews had been conducted, an initial Rapid Assessment Procedure (RAP) sheet [43, 44] was developed (Additional file 2). Interviewers subsequently used the RAP sheet to summarise each interview as relevant to the developing analysis, to help us to think about how the new data related to the existing analysis and to share findings with other members of the research team. Informed by these oral analysis discussions, one of the co-authors (RT) undertook a detailed thematic analysis of the verbatim transcripts. This involved close reading of the transcripts and making annotations to highlight items or quotes that were potentially interesting or significant. Data from each participant at each site was collated into a table divided into three sections: (1) theme name, which identified potential main themes; (2) sub-themes, which explored subordinate issues relating to the main themes; and (3) description of the theme, which gave an explanation and/or and descriptive examples from the transcripts. The preliminary themes were then compared back to the transcripts to assess whether they were a good reflection of the interview data, to identify disconfirming evidence and to explore any additional issues that had not been addressed in the initial themes. This verification process also included detailed discussions within the research team, during which the relevance of each theme was established and the themes/sub-themes were further refined. Additionally, “pen portrait” style summaries [45] of each site’s journey through the trial were created to aid comparisons across sites, helping to identify commonalities and divergences which could further inform the analysis.

Data collection and analysis was sensitised by Normalisation Process Theory (NPT), which has been widely used to study implementation in healthcare contexts, including in trial process evaluations [42, 46, 47]. NPT incorporates four constructs: coherence (how people make sense of the trial); cognitive participation (whether people are willing and able to buy-in to implementing the trial); collective action (people’s ability to take on the work needed to implement the trial); and reflexive monitoring (people’s reflection on the benefits and costs of the trial) [42].

Towards the end of analysis period, several of the oral analysis discussions focussed more explicitly on exploring the emerging findings in the context of (a) existing research on trial conduct and (b) Normalisation Process Theory (NPT). To help with this process a coding framework was developed (Additional file 3) which linked the research questions for the qualitative study to each of the NPT concepts and components, following the process set out by Murray and colleagues [48] for using NPT in feasibility studies to optimise trial parameters. One of the co-authors (RH) then went through a process of synthesising and triangulating insights from both the site summaries and the thematic analysis by mapping them against the tailored NPT framework, following a similar process to that adopted by Bamford and colleagues in their NPT-informed analysis of an implementation evaluation [49].

### Ethical considerations

The original FORVAD trial was approved by Northwest Greater Manchester Central Research Ethics Committee (reference number 18/NW/0682). The qualitative sub-study was approved via a substantial amendment to the protocol on 10 February 2021.

All participants in the qualitative study were referred to by an identification number and all trial sites were assigned a letter (e.g. Site A etc.) to protect anonymity. Participants are described in reports based on their role only and are not linked to the trial site to reduce the risk of the participant being identified. All quotes used in reports have been redacted where necessary to protect the anonymity of participants and sites.

## Results

### Participants

Eight out of the seventeen sites approached to take part in the qualitative study agreed to take part, including four that were open to recruitment and had randomised at least one participant, two that were open to recruitment but had not yet randomised a participant and two that were still in set-up when the trial closed (Table 1). Two sites were not able to confirm continued capacity and capability for the study amendment and so declined to take part in the study. Seven sites did not respond to recruitment emails or follow-up recruitment emails; no further efforts were made to recruit these sites once we had successfully recruited at least two sites in each of the different categories: in set up; open; recruiting.

**Table 1:** Sites participating in the qualitative study

*Site code*	*Number of interviews*	*Site status*
*A*	*5*	*Open and randomised*
*B*	*3*	*Open and randomised*
*C*	*2*	*Open but didn’t randomise*
*D*	*2*	*Open and randomised*
*E*	*1*	*Open but didn’t randomise*
*F*	*2*	*Open and randomised*
*G*	*2*	*Set-up*
*H*	*1*	*Set-up*

Eighteen interviews were conducted: four with surgeon PIs, five with other participating surgeons and nine with research nurses or study coordinators. Given the small number of participants per site, to maintain confidentiality we have labelled quotes with participants’ professional background only.

### Findings

The thematic analysis generated four overarching themes related to recruitment challenges: (1) individual vs. community equipoise; (2) trial set-up and delivery; (3) identifying and approaching patients; and (4) timing of randomisation.

### Individual vs. collective equipoise

There was widespread support for the aims of the FORVAD trial amongst participating surgeons because they recognised community equipoise between the two interventions—i.e. a lack of clear evidence on which to base the decision to use ACD over PCF, or vice versa. FORVAD was therefore viewed as a worthwhile study that would help to improve clinical decision making for this condition:

“It really is something that we don’t know and we as clinicians make the decision between anterior and posterior surgery all of the time without really knowing which is the best option.” (Surgeon)

“I like the question that has been posed by the study - is foraminotomy or anterior cervical discectomy better? Because that’s a constant argument, in the medical, I would say, community” (Surgeon)

Despite this recognition of community equipoise, however, and their own ability to deliver both interventions, many interviewees highlighted that preferences existed amongst their colleagues and/or at other sites. This was often linked to the fact that many centres have a tradition of performing interventions in a particular way, which then affects the training and experience acquired by new surgeons:

“Different units tend to have different views, and they’re very strong views on this. Some people will do everything from the back, some people will do everything from the front, and that is how they, and their trainees are trained that way and that is how things work.” (Surgeon)

“Surgery’s basically an apprenticeship, so you learn from your training person who then was trained by the other people in the unit. So all of these skills get handed down through generations of surgeons. And unless there’s new people coming into the centre, it won’t necessarily change” (Surgeon)

Although a preference does not necessarily prevent an individual from recruiting participants to a trial, interviewees suggested that in the case of FORVAD these preferences did impact trial engagement—this was sometimes referred to as having or not having equipoise:

“You need to find surgeons who have equipoise, you know, and I certainly had it and a lot of the people who were excited about this trial had it. But if you were to go to a centre where the tradition is to do everything one particular way, there won’t be any equipoise. So I don’t know if [hospital name] agreed to participate but that’s an example of a centre where they do everything through the back of the neck and they won’t have any equipoise, so they wouldn’t want to participate.” (Surgeon)

My personal reason, and anecdotal from my colleagues, is a simple thing, why we are struggling to recruit, with regards to the training we had and what, all we do, there was no ambiguity in choosing an foraminotomy or an ACD. So there’s no, what is that word, [equipoise or…?] Yes, exactly, equipoise. [Laughs] So there is no [laughs] equipoise. Because from our training, from my training, particularly, if I see a patient, there’s no equipoise at all, I just go for an ACD. (Surgeon)

Some interviewees also suggested that individual equipoise could be impacted by the differential risk profile of the two interventions—with ACD perceived as having a risk of rare but more serious complications than PCF—and the fact that ACD is a more complex procedure that might be more difficult to perform for an inexperienced surgeon.

“In terms of safety profile both are very, you know, equally similar. But if it does go bad in the front, it goes bad disastrously.” (Surgeon)

“I would say the front of the neck takes longer to get to grips with.” (Surgeon)

In addition to a general preference for one or other procedure, interviewees also discussed the fact that a surgeon might consider that a specific procedure—which might not necessarily be the one that they have a more general preference for—was best for an individual patient. This could be for various reasons, both clinical and non-clinical:

“If you have a disc and an osteophyte which is compressing mainly from the front, to then consider an operation from the back sometimes can be difficult to justify, even for the most academically minded person.” (Surgeon)

“The only reason I’ll go through the back of the neck is if it’s quite low on the neck whereby if it’s too low in the neck your collarbone gets in the way to get into the spine, whereas at the back of the neck you can go all the way down through the spine.” (Surgeon)

“If they utilise their voice professionally, so if they’re a singer for example, then they may be more swayed to having an operation from the back of the neck.” (Surgeon)

Overall, there appears to have sometimes been some conflict between community and individual equipoise at some of the FORVAD sites—i.e. despite recognising the general uncertainty around the best choice of interventions, an individual surgeon might often feel that one procedure would be better than the other in specific situations. This may have affected how many patients were deemed suitable to take part in the trial, even if they technically met the eligibility criteria:

“Quite a few people, they said, “No, they need to have an anterior or a posterior”, and that’s fine, I mean, we can’t argue with that, can we, we’re not the surgeons, we have to accept that that’s the case. But it left only a small number of people who they thought were suitable, that there was equipoise in.” (Research Nurse)

### Trial set-up and delivery

Obtaining research governance approval was a protracted process at many FORVAD sites, and some interviewees reflected on how this prolonged set-up period led to a loss of momentum and feelings of frustration for trial staff:

“To start recruiting in [site name] that was a long time, like eight, nine months, and the information had actually gone from the minds of the surgeons, and gone from the mind of the trainees, so it was, wow - very, very challenging” (Research Nurse)

Support from dedicated research support staff and previous experience of obtaining governance approval were identified by many interviewees as important factors in successfully navigating the set-up process. Research support was also invaluable in ensuring that trial processes could be incorporated into the usual clinical workload, as surgeons often lacked the time and expertise to complete all the planning and paperwork associated with the trial:

“You see, I would be lost without a research nurse, that’s the first thing to say. I think, by and large, the paperwork and the minutiae of things and the actual X, Y and Z and the process and all of that, that is where they keep you right. And I think that is very important” (Surgeon)

If research support was not available (in the following quote the interviewee is referring to a research administrator to support the research nurse, but in other sites research nurse availability was also an issue), it was difficult to set up and sustain the trial.

“We can’t participate because there is no support for the CRN. Unfortunately they have created a huge bureaucracy that is necessary to start the study, but there is no support to do it” (Surgeon)

Overall, it appears that having an experienced team working together effectively was perceived by interviewees to be a crucial factor in successfully implementing neurosurgery trials. Key to this appears to be the ability of the team to go beyond their usual remit to make trial processes “work”—for instance, taking on extra responsibilities such as site initiation visits, or coming in to work on a day off as described by this interviewee:

“Most of the times they would have surgery on a Friday which means Saturday I was not going to be working but then because of the following day post-op …I had to come to work so that I could come and just do that so that we don’t miss the patients as well and so that we don’t miss the paperwork as well.” (Research Nurse)

Another way that interviewees described going above and beyond standard working practices is by accessing further support and learning through both formal and informal networks (for example WhatsApp groups and social media pages), as in this example:

“…Some trials I work on we’ve got a WhatsApp group, so when I worked at *[hospital name]* you could offer support or if you worked on a specific trial; some Trusts that work seven days a week, there was always someone there to ask a question….one trial I’m thinking of, they did set up the WhatsApp group themselves at co-ordinating centre” (Research Nurse)

### Identifying and approaching patients

Interviewees at sites that struggled to recruit often described difficulties in identifying enough eligible patients to approach about the trial. Although some perceived this to be due to a lack of suitable patients being seen at the site, others suggested that suitable patients were in fact being seen in clinic, but surgeons may have been failing to identify and approach them:

“After we’d opened, those were the meetings we would go to, to say, “Come on, guys, you told us there were loads of patients, you told us we were going to do alright. And now I’m literally squeezing these names out of you of these people who’ve been to clinic, who would seem to be suitable for the study” (Research Nurse)

“They just, just never seem to be able to keep it in the top of their heads. And we then also would get the lists and go through them, and then say, “Oh, what about Mrs X? You know, you saw her in clinic last week, don’t you think she’d be suitable?”, “Oh, yeah, yeah, she’d be suitable, yeah” (Research Nurse)

At some sites there appeared to be an issue in engaging clinicians beyond the PI in recruitment.

“We did have other people delegated on the study but then they change over, so it’s having to keep up with the changes. So we only really had our PI who was obviously a consultant and permanent; there wasn’t many other people to ask if they could help with the study. And all of the other surgeons are PIs on other studies and they don’t like to cross-collaborate, they do what they do, they don’t like to dip into each other’s studies” (Research nurse)

These issues appear to have been most prominent at sites which took an ad hoc approach to identifying potential participants, with patients only being considered if the clinician whose clinic they had been allocated to was involved in the trial. The site that recruited most successfully took a more structured approach, identifying potentially eligible patients from referrals and directing them to a participating surgeon’s clinic, where research support staff would be on hand to facilitate recruitment:

“Instead of seeing suitable patients randomly, what we did was we pulled all the suitable patients to a specific trial clinic” (Surgeon)

As well as this variation in the process for identifying eligible patients, there were also differences between sites in terms of who initially approached patients about the trial. Some interviewees felt it was important for the initial conversation to be with the treating clinician, whereas others felt this could appropriately be done by a research nurse:

“Yeah, I would say a research nurse can very well approach us, because a research nurse has most of the information or all of the information, I would say.” (Surgeon)

Another source of variation between sites that may have affected ability to identify suitable patients was the length of their waiting lists, and particularly any difference in waiting times between the two trial interventions. According to one interviewee, lengthy waiting lists for either procedure raised the likelihood of the operation being performed at a private hospital, leading to participants being withdrawn from the trial since the private facility was not a trial site.

### Timing of randomisation

Although trial participants could be consented and registered on the trial up to 6 weeks before surgery, randomisation took place on the day of surgery, meaning that both patients and the clinical team did not know until the day of surgery which of the trial interventions the patient would receive. This appears to have created several logistical challenges, for instance this interviewee describes the practical challenge of completing all the trial procedures on the day of surgery when the patient was due in theatre early in the morning:

“Then the issue about consent on the day with the way people coming in on the day of surgery. In any case, having to be ready in the coming in at seven, going to theatre of their list by nine, then going through everything again. I think there’s going to be a real challenge” (Research Nurse)

Randomisation on the day of surgery also meant that the surgeon and theatre team had to be prepared to deliver both procedures, as they did not find out the allocation until shortly before the patient arrived in theatre. This did not always fit well with established processes for planning and managing theatre lists, as explained in this quote:

“Having to do it short notice on the day, of course, because you would have had to, you know. I can just imagine the bureaucracy….” Okay, what operation this patient having?” You then end up putting “Well…they might be having one or two” and then going to the nursing staff. It’s tough to access an ACD opposed to a PCF. There are different instruments” (Surgeon)

As well as causing logistical issues in theatre, randomisation on the day of surgery also created difficulties with the surgical consent processes at some sites. Surgical consent tends to be taken at an outpatient clinic some time prior to the day of surgery and then reconfirmed on the day of surgery. Because the randomised allocation was not known at the time of the consent clinic, FORVAD participants had to be consented for both procedures, with consent for their allocated procedure then being confirmed on the day of surgery once the randomisation had been done. Taking surgical consent for two procedures put pressure on the time available for pre-op clinics, as explained by this surgeon:

“Consent slots should last 30 minutes, and if the study’s involved it will require additional 15 or 20 minutes…and also more paperwork. So it’s left everything I would say half done” (Surgeon)

Some interviewees also struggled with taking surgical consent for two procedures, one of which the patient would not end up having. There was a perception that it could be confusing for patients or made it difficult to properly explain the risks and benefits of the procedure the patient would have, as exemplified in this quote:

“It might have been better to randomise them in advance. Because, you know, it’s the issue of consent, you know, what risks to warn a patient about and there’s no denying the risks are different between the two operations.” (Surgeon)

Randomisation of the day of surgery potentially posed ethical as well as practical issues for participating surgeons. Although patients were consented for both procedures in advance, they only confirmed their consent for the actual procedure they had on the day. This appears to have caused concern, with some interviewees mentioning a recent legal ruling in which a hospital had been found to be negligent in a case where a patient had been given significant new information on the day they underwent surgery. Although some interviewees did not appear to view this as a relevant risk in the FORVAD trial, others perceived there to be at greater risk of being seen as liable if something went wrong with the procedure:

“You put the patient on straps for foraminotomy and they actually consented to ACD from the day something goes wrong. This patient was actually listed for this operation, but actually had that operation. If it goes wrong it presents its own set of problems in itself in that way.” (Surgeon)

Overall, the timing of randomisation appears to have made FORVAD recruitment difficult in several ways. Some sites were not prepared to take part in the trial at all because of it, and at some sites that did participate it appears to have contributed to delays in opening because site teams had queries and concerns about the process.

## Discussion

### Understanding FORVAD’s recruitment challenges

The thematic analysis outlined above suggests that there was no single issue that made recruitment to the FORVAD trial problematic. Rather, as has been found in other studies [20, 32], there were a range of inter-connected factors that combined to impede recruitment. Notably, these factors appear to have materialised to a greater or lesser extent at different sites; no one site described experiencing every issue to the same degree, but all experienced some of them to some extent. Individually, none of these issues appear to have been insurmountable, as sites that recruited successfully described experiencing many of the same issues as those that failed to recruit. Likewise, these results suggest that some facilitators, whilst identified as being important, may be necessary but not sufficient for successful recruitment. For example, some surgeons interviewed at sites that failed to recruit reported being supportive of and engaged with the research question and were willing and capable of delivering both interventions. Conversely, surgeons at sites that did recruit described struggling to convince all their colleagues of the benefits of both interventions.

In this context, drawing firm conclusions about how, why, and to what extent the factors identified in the thematic analysis impacted on recruitment is difficult. Normalisation Process Theory (NPT) is helpful in this regard, as it provides a structured framework for understanding challenges of implementation and integration [48]. Here, we consider the results of the thematic analysis in the context of the four NPT core constructs (coherence, cognitive participation, collective action and reflective monitoring), identifying key aspects of the FORVAD trial that challenged sites’ ability to embed the trial processes in their day-to-day practice.

### Coherence (meaning and sense making by participants)

Overall, there appears to have been widespread support for the aims of the FORVAD trial, and participating surgeons felt that the research question was important and that the results would have influenced clinical practice. The differences between the two procedures were well understood and surgeons discussed the relative risks and benefits of each, identifying certain characteristics or circumstances that would make one better than the other for specific patients. However, participating surgeons also felt that there were some patients for whom either procedure could be appropriate and that there is a lack of evidence as to which procedure is better in these circumstances. The trial eligibility criteria were generally felt to be appropriate and identified those patients for whom it was unclear which procedure would be best (i.e. where there is collective clinical equipoise).

There does not appear to have been any other significant issues with the FORVAD trial in terms of communal or individual specification. Site staff did not report any concerns around information provision, training or understanding of the trial aims or benefits, and generally both surgeons and research support staff appeared to be clear of their roles and how they fit with the trial overall. However, one issue that did appear to present a significant challenge in terms of coherence was the timing of randomisation. Randomising on the day of surgery conflicted with a general move towards earlier surgical consent because of medico-legal concerns and challenged surgeons’ clinical relationships with patients by removing their opportunity to discuss the specific procedure the patient would have at their pre-operative out-patient clinics. Although none of the surgeons interviewed specifically identified this as a key factor in their recruitment problems, it clearly limited their ability to make sense of the trial in the context of their legal and clinical obligations. The potential impact on surgeons’ willingness to recruit and enthusiasm for the trial should therefore not be discounted, particularly for those surgeons who may have been less engaged to begin with (and who are likely to be un- or under-represented in our interview data).

### Cognitive participation (commitment and engagement by participants)

Commitment and engagement emerged from the thematic analysis as a crucial factor in sites’ ability to set up and recruit successfully to the FORVAD trial. This is not to say, however, that commitment and engagement was necessarily lacking at sites that struggled with recruitment, as all interviewees described being enthusiastic about the trial and keen to take part. Rather, it appears that the considerable logistical challenges of delivering a surgical trial could (in some cases) be overcome by an elevated and sustained level of commitment and engagement. Conversely, these challenges appear to have had the effect of dampening enthusiasm at some sites, and it may be that being moderately committed and engaged with the trial initially was not sufficient to maintain the level of motivation required to push through the numerous barriers to operationalising the trial in practice. The importance of having an engaged, experienced and motivated site team who are prepared to “go the extra mile” to deliver the trial clearly cannot be underestimated. Two types of team were particularly important to trial implementation—firstly, the multidisciplinary research delivery team, involving the PI and research nurses and administrators. As in previous studies, research support was sometimes difficult to access at sites [50]. When designing future trials, therefore, consideration should be given to making the trial as easy to set up and deliver as possible (taking into account the specific implementation challenges discussed under collective action below), so that site staff can focus their limited time and energy on recruitment. The second type of team was the wider surgical team, and the ability of the PI to enrol their surgical colleagues in trial recruitment and delivery.

Another important factor to consider in terms of cognitive participation is legitimisation, and whether there was sufficient equipoise amongst clinical staff at sites. Although equipoise did emerge as a key issue in the thematic analysis, this was largely in the context that some hospitals and/or surgeons would not consider taking part because of strong preferences for one of the trial interventions over the other. This will have limited the number of sites willing to participate, and potentially the number of participating surgeons at sites that did take part. However, it is unlikely that this was the key factor limiting recruitment, as sites that recruited successfully did so with only a small of number of surgeons delivering the trial interventions, and the total number of sites that opened or were in set-up should have been sufficient if those sites had recruited at the same rate as the more successful sites. Furthermore, in line with their general support for the aims of the trial and agreement that there is collective clinical equipoise, the surgeons interviewed largely appeared to have sufficient individual equipoise to deliver the trial successfully. Likewise, in contrast to research on other surgical trials that found equipoise to be the key factor limiting recruitment [51], the research nurses and other research support staff interviewed for FORVAD did not report any significant issues with surgeon preference hampering their ability to recruit. Fewer eligible patients declined the trial (*n* = 15) than were willing to take part (*n* = 27) [11] suggesting that when surgeons did talk to patients about the FORVAD trial, that they were able to communicate equipoise successfully enough for patients to consent to taking part (although recordings of recruitment conversations would have been useful to assess this further). The key limiting factor appears to be not the ability of the team to communicate equipoise in a trial recruitment conversation but rather the timely identification of eligible patients so that the recruitment conversation could take place.

### Collective action (i.e. the work participants do to make the trial function)

The thematic analysis, in particular some of the issues explored under the *trial set-up and delivery, identifying and approaching patients* and *timing of randomisation* themes, suggests that interactional workability (how the trial was operationalised and its compatibility with existing work practices) is likely to have been one of the most important factors affecting recruitment to the FORVAD trial. The process for screening and approaching patients, the timing of randomisation, fitting follow-up visits into standard care pathways, the length of waiting lists and the availability of theatre facilities and/or use of private facilities were all important aspects of operationalising the trial. Importantly, sites that recruited successfully were either not affected by these issues (for instance waiting lists were relatively short because the site had access to theatres dedicated to spinal procedures, or follow-up visits happened to fall within their usual clinical pathway), or described putting processes in place that overcame them (e.g. identifying potentially eligible patients from referral letters and directing these patients to dedicated clinics run by participating trial surgeons). Conversely, sites that recruited less well described having issues with some or all these activities. For example, one site experienced a significant delay in opening because of concerns about randomisation on the day of surgery, and another interviewee described arranging follow-up visits that were outside of the usual care pathway taking up time that could otherwise have been spent screening. Even in cases where interviewees felt their recruitment problems were predominantly due to not seeing enough eligible patients, it was apparent that the site may in fact have simply failed to identify those patients because of the lack of a structured process for screening referrals.

In terms of skill set, workability and contextual and relational integration, the thematic analysis highlighted the importance of site clinical teams and research support working together effectively, and the crucial role played by research nurses. In addition to the importance of having surgeons with individual equipoise and an experienced and motivated team who are willing to “go the extra mile” (as discussed above), learning from each other and establishing support networks appeared to be particularly valuable for many interviewees.

### Reflexive monitoring (i.e. participants reflect on or appraise the trial)

The potential for reflexive monitoring of the FORVAD trial was limited to some extent by the circumstances in which the trial closed. In particular, three interlinked factors are likely to have affected participants’ ability to reflect on and appraise the trial: [1] the short length of time many sites were open limited their opportunity to reflect on the trial processes; (2) the impact of Covid-19 in the last few months of the trial meant clinical services were severely disrupted, making it difficult to reflect on the trial itself; and (3) the early closure of recruitment means the research question has not been answered, so participants are not able to reflect meaningfully on the outcome of the trial and the potential for changing clinical practice. Notwithstanding this, however, the thematic analysis does provide some indication of reflexive monitoring and suggests some tentative conclusions which may be of value for future research in this area.

### Implications for future research

The surgeons interviewed were generally still supportive of the aims of the FORVAD trial and felt that this is still an important clinical question to be answered. In terms of individual appraisal, and in particular the impact of the trial on individuals and their work environment, there were some concerns about the amount of time the trial required and how difficult this was for surgeons to fit into an already full clinical schedule. It does not appear, however, that FORVAD was uniquely challenging in this regard, and although there were some aspects of the protocol that created additional work this does not appear to have been a significant factor that limited recruitment. It is likely, therefore, that this concern is largely applicable to trials in surgery in general, rather than being a specific issue for FORVAD. It does suggest, however, that it is important to take into account the context in which site teams are working when designing surgical trials, and to ensure that site staff can focus their limited resources on screening and recruitment as much as possible [31, 52]. Assessments and data collection should be kept to the minimum required to answer the research question and visits should be aligned as far as possible to standard care pathways to minimise the additional work required at sites.

In line with this, the thematic analysis suggests that sites only had limited ability for reconfiguration and adapting the trial procedures based on their experience. The nature of clinical trials means that some inflexibility is inevitable, as there needs to be some consistency across sites. However, consideration should be given when designing future trials to identifying aspects of the protocol where flexibility can be allowed without significantly affecting scientific validity, so that sites can adapt the trial to their working practices as much as possible.

As with other surgical studies, surgeons in FORVAD had preferences relating to the two procedures under investigation. Training may help surgical teams to overcome preferences and communicate collective equipoise [4, 32]. Additionally, expertise-based designs have been proposed as a route to addressing recruitment issues where surgeons have preferences [53]. In an expertise-based design, surgeons who recognise collective equipoise could participate in the trial even if they did not feel personally able to deliver both interventions. Any benefits, however, would need to be weighed up against the additional logistical challenges introduced by an expertise-based design [54] particularly in the context of the interactional workability challenges experienced in FORVAD.

Additional feasibility work or a smaller-scale external pilot could potentially have enabled some of the issues identified here to be addressed prior to a full trial. However, feasibility work can sometimes over or underestimate future recruitment success, and external pilots add time into what is often already a lengthy evaluation process [55].

By adopting a rapid qualitative analysis approach, our study was able to quickly gather and interpret information about barriers to successful trial conduct, identifying aspects of the trial design and delivery that could potentially have been modified to facilitate recruitment. Although rapid approaches are starting to be used more frequently in evaluation and appraisal research [43, 56, 57], they have been less widely used in trials [58]. More widespread use of rapid qualitative approaches might enable trial teams to more quickly identify and address barriers to successful trial conduct, reducing the need for extensions, early closures and associated research waste.

### Strengths and limitations

Key strengths include the rapid analysis methods which enabled a rigorous approach within a short timeframe, and the successful recruitment from our three categories of site (recruiting; open but not recruiting; in set up). The use of a theoretical framework was an important factor in the success of our rapid qualitative approach. NPT has often been used to study the uptake and adoption of new healthcare interventions within the context of a clinical trial [46, 48, 59]. It has more rarely been used to study the trial itself as the intervention of interest, although Murray and colleagues [48] have suggested a framework for using it to assess the feasibility and optimise the design of complex intervention trials, and there have been some examples of it being used successfully in this way [60, 61]. We found that it had considerable value for our analysis, as it provides a structured analytical framework with a focus on trial implementation [48, 62]. Using a theoretical “lens” to interrogate the thematic analysis, particularly in conjunction with the site summaries, allowed us to quickly pinpoint the factors that were most likely to have hindered the effective implementation of trial procedures at sites. Future work could usefully synthesise studies that have applied NPT to trial implementation to draw out similarities and differences and with the aim of pre-empting obstacles in future trials.

Despite these important strengths our study also had some limitations, primarily related to its timing. Because the qualitative evaluation was only added to the trial after the decision was reached to close it, we were only able to retrospectively identify the factors that made recruitment challenging. If the qualitative research had been planned at an earlier stage it could have enabled an iterative, formative evaluation to improve recruitment and perhaps avoid the need to close the trial early. Additionally, the timing of the qualitative study and the limited time available for fieldwork, as well as the Covid-19 restrictions in place at the time, limited our data collection to remote interviews. Although these interviews elicited rich and useful data, confidence in our findings would have been strengthened if we had been able to combine the interviews with observational fieldwork during recruitment.

The timing of the study also meant that our sample was limited to sites that were already in set-up or open, and to site staff that had chosen to participate in the study. This meant that we were unable to incorporate the views of sites and surgeons that had chosen not to participate, and it is likely that our interviewees skewed towards those who were more positive about the trial aims and more engaged in its delivery. Although the qualitative researchers conducting interviews and analysis for the qualitative sub-study had not previously been part of the trial team, members of the trial team were involved in recruiting sites to participate in the qualitative study, and the qualitative researchers were employed within the Clinical Trials Research Unit managing the FORVAD trial. This may have impacted what participants chose to share with the qualitative researchers about their views and experiences of the FORVAD trial. One way to address this in future studies would be to offer site staff the option of providing anonymous feedback, e.g. via a survey; such an approach might also broaden the range of staff who contribute, including individuals at sites that choose not to take part in the qualitative study, allowing an element of triangulation in the analysis.

The timing of the qualitative research also limited our ability to explore the experiences of participants involved in the FORVAD trial. It was not possible to observe recruitment conversations or interview those who had declined the trial because recruitment had already finished when the qualitative fieldwork took place. We did aim to interview trial participants; however, a combination of factors (approvals for research staff; small pool of participants; low response) meant that we were only able to complete two patient interviews during the fieldwork period. Although these interviews provided some useful insight into the participant perspective, particularly around their experience of the intervention, they represent only a very small sample and only of those who chose to participate and hence made a limited contribution to our understanding of why recruitment was difficult (and have therefore not been included in the analysis and discussion presented here). Incorporating the participant experience more thoroughly would have provided a more nuanced and rounded understanding of the recruitment process.

## Conclusion

The results of this qualitative study suggest that overall there was support and enthusiasm for the FORVAD trial and for trials in this clinical area in general. Interviewees confirmed that community equipoise is present; however, individual equipoise appears to have been an issue both at the overall site and individual surgeon level. The extent to which individual equipoise affected recruitment is not clear, and all participating sites had some surgeons who were willing and able to deliver both procedures. However, the lack of more widespread individual equipoise amongst recruiters limited the number of sites and surgeons who would take part and may have made it more difficult for participating surgeons to engage their colleagues in the trial.

In addition to this, the difficulties in recruiting appear to have been predominantly caused by a combination of interlinked factors related to the interactional workability of the trial. Randomisation on the day of surgery appears to have been an issue in various ways, slowing down set-up at some sites and limiting the number of surgeons or sites willing to take part. The process for screening and approaching potential participants also appears to have been a crucial factor, and a structured approach to identifying and approaching eligible patients allowed for successful recruitment even in sites with limited numbers of eligible participants and participating surgeons. Delivering the trial successfully also required individuals and teams to do more than just their day-to-day role. Support, engagement and motivation are crucial for this, and elements of the protocol that were hard to incorporate into the usual clinical pathways are likely to have made this harder to maintain.

Importantly, there does not appear to have been one specific factor that made FORVAD recruitment difficult at some sites and more successful at others. Rather, there were a range of factors that facilitated or impeded recruitment to a greater or lesser extent at each site. Some of these issues can potentially be addressed in the design of future trials; for instance, encouraging sites to adopt a structured process to identify and approach potentially eligible patients and avoiding randomisation on the day of surgery unless there is a compelling scientific justification. In addition to these specific suggestions, however, consideration should be given when designing future trials to ensuring the protocol is as flexible as possible, thus allowing sites to address specific interactional workability issues at a local level. Rapid, theoretically informed qualitative research conducted during the trial design, set-up period, and pilot stage would help to achieve this, identifying key barriers and ensuring trial processes support interactional workability as far as possible, thus maximising the opportunity for sites to recruit and addressing issues pertaining to individual equipoise in complex research environments.

### Patient and public involvement (PPI)

The FORVAD trial had PPI throughout its duration, from the grant application stage through to trial completion. Mr Martin Gledhill served on the TMG and Dr Catherine Pinnell was a member of the TSC.

Both PPI representatives attended trial oversight committee meetings and were actively involved in trial discussions, providing valued opinions and ideas from a patient and public perspective. PPI input also fed into the protocol design, the drafting/reviewing of participant information resources, the interpretation of the results and the write-up of the final report.

### Principal investigators

Thank you to all of the PIs who participated in the trial: Mr Yahia Al-Tamimi (Sheffield Teaching Hospitals NHS Foundation Trust), Mr Neil Buxton (The Walton Centre NHS Foundation Trust), Mr Nicholas Haden (University Hospitals Plymouth NHS Trust), Mr Nitin Mukerji (South Tees Hospitals NHS Foundation Trust), Mr Ravindra Nannapaneni (Cardiff and Vale University Health Board), Professor Marios Papadopoulos (St George’s University Hospitals NHS Foundation Trust), Mr Anantharaju Prasad (Lancashire Teaching Hospitals NHS Foundation Trust), Mr Ivan Timofeev (Cambridge University Hospitals NHS Foundation Trust), Mr Christos Tolias (King’s College Hospital NHS Foundation Trust), Mr Senthil Selvanathan (Leeds Teaching Hospitals NHS Trust) and Mr Nitin Shetty (Whittington Health NHS Trust).

We would also like to thank all other members of the local research teams at each and every one of the FORVAD trial sites for their valued contributions to the set-up and delivery of the trial.

### Supplementary Information

Additional file 1

Additional file 2

Additional file 3

Additional file 4

## Data Availability

The dataset supporting the conclusions of this article is available from the Clinical Trials Research Unit of the University of Leeds on reasonable request. Access to the data is subject to approval and a data-sharing agreement due to participant confidentiality.
