# Supplementary material for: Rapid qualitative analysis of recruitment obstacles in the FORVAD (Posterior Cervical Foraminotomy surgery versus Anterior Cervical Discectomy surgery in the treatment of cervical brachialgia) randomised, controlled trial
Source: Trials. 2024 Aug 17;25:546. doi: 10.1186/s13063-024-08391-4 (PMC11330054; doi:10.1186/s13063-024-08391-4)
Supplement: Supplementary file 1 — Additional file 1 [file 13063_2024_8391_MOESM1_ESM.docx]

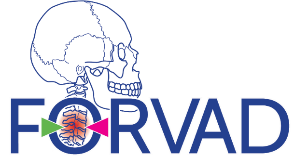


**FORVAD Interview Sub-study**

**STAFF INTERVIEW TOPIC GUIDE (Professionals)**

**Version 1.0**

**FORVAD: Interview Topic Guide for Professionals**

***Note:*** *The interview schedule is developmental. The questions will need to be tailored to the specific answers of each interviewee. The interview schedule given here is therefore a general topic guide for the one-to-one qualitative interviews. Not all questions will be appropriate e.g. for sites that did not recruit participants to FORVAD*

### Welcome and Introduction

Introduce self and project. Discuss confidentiality and recording. Ask if any questions. Confirm ongoing informed consent.

**Re-cap of Research and Plan for Interview**

Brief re-cap on the aims and purpose of the interview and explain what will happen.

***The following questions need not be covered in this particular order but rather the interview should flow as freely and naturally as possible. The interviewer will prompt as appropriate with phrases such as ‘can you tell me a little more about that’, ‘can you give me an example of that’, ‘how did/do you feel about that’.***

### Question: Can you tell me a bit about yourself from the point of view of your career?

### Views on FORVAD - overview

- What point did you hear about it?
- Why did you decide to take part/not to take part?
- What has been your role on the study?
- How do you feel about the research question now?
  Is it important?
  Did you have any reservations about the FORVAD trial?
  - Outcome measures
  - Inclusion criteria
  - Consent process (timing of consent)
- How did you feel about FORVAD closing early?
  - Were you surprised that FORVAD was unable to recruit to target?

**Setting up FORVAD**

Can you tell me a bit about the process of setting up FORVAD?

- R&D and other site approvals
- Engaging colleagues
- Training/SIV

**Views of trials in cervical brachialgia and Neurosurgery**

- Do you think randomised controlled trials are the right way of addressing this research question?
- Do you think the results of FORVAD would have led to changes in clinical practice?
- Have you taken part in any other randomised controlled trials? Can you tell me a little about those?

### Views of cervical brachialgia

- Are there different groups of people who have cervical brachialgia?
- What symptoms seem to bother people the most?
- What factors did you take into a/c prior to FORVAD when deciding whether to list people for surgery – which of these were the most important?

**Surgical approach (Anterior Cervical Discectomy and Posterior Cervical Foraminotomy)**

- What was your surgical approach for cervical brachialgia pre-FORVAD?
- What factors did you take into a/c prior to FORVAD when deciding on surgical approach
- What are Anterior Cervical Discectomy and Posterior Cervical Foraminotomy like as an operation – easier/complex compared to other procedures that undertake – what sort of complications etc
  - Technical difficulty of procedure (risk of injury to vessels, Operative planes – whether well visualised
  - Any specific/new equipment
  - What are outcomes like from these operations?
- How long have they been doing surgical approach – how many do they do a year – how has their activity changed over time
- How much variation in practice is there in relation to surgical approach for cervical brachialgia at this site

### What helped you perform your role in FORVAD

- What has made it more difficult?
- How has the FORVAD trial fitted with your normal working methods for this patient group?
- How has the FORVAD trial fitted with established clinical pathways?
- What impact has recruiting patients for the study had on the day to day running of the clinic?

**Changes during FORVAD**

What FORVAD activities have they participated in (site initiation visit, teleconferences etc)?

- Did you make any changes at this site to make FORVAD fit more easily into pathways
- Did you make any changes to the way you recruited patients or to their pathway during FORVAD with the aim of improving recruitment, management to protocol or follow up?

### Experiences of recruitment

- How has recruitment been and how do you go about recruiting patients?
  *Explore previous experience of research and recruitment*
- *What factors have helped/hindered recruitment?*
- Have there been any patients who met the inclusion criteria but you felt were not appropriate for the study?
- From your perspective, what are the facilitators and barriers to getting people/patients engaged in the study?
  *Explore facilitators & barriers from staff and patient perspectives*
- Who has taken the lead in explaining the study to patients and how have (they) gone about it?
- How else could we support recruitment in future trials?

**Experiences post recruitment**

- How did people respond when received allocation?
- Any issues with delivering treatments to protocol?

**Experiences of delivering treatments**

- Who is involved in the care of people with cervical brachialgia – surgeon – but who else (anaesthetist, nurses, physio etc)
- How does surgery in FORVAD compare with normal clinical practice at this site
  - (type of surgeon, time to operation)
- What has been happening externally to the trial that might impact on surgery for cervical brachialgia now or in the future
- Changes to how surgery conducted – what debates are there currently in terms of cervical brachialgia clinically - detail of suturing, approaches to the actual surgical techniques etc.

Has participation in FORVAD altered how you see surgery for cervical brachialgia? How?

**Measuring eligibility and outcomes**

- Follow up – experiences of this
  - Experience of inviting people to attend
  - have there been any surprises?
- Experiences with trial eligibility & outcome measures

### Anything not covered?

### Anything else you’d like to add about your experiences of the study?

**Closing and Thanks**

Conclude the discussion and thank the participant for their time and contribution.
