# Supplementary material for: Rapid qualitative analysis of recruitment obstacles in the FORVAD (Posterior Cervical Foraminotomy surgery versus Anterior Cervical Discectomy surgery in the treatment of cervical brachialgia) randomised, controlled trial
Source: Trials. 2024 Aug 17;25:546. doi: 10.1186/s13063-024-08391-4 (PMC11330054; doi:10.1186/s13063-024-08391-4)
Supplement: Supplementary file 2 — Additional file 2 [file 13063_2024_8391_MOESM2_ESM.docx]

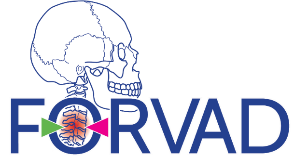


**FORVAD Staff Interview RAP Sheet**

| **BACKGROUND** | |
| --- | --- |
| **Job/role** | **Involvement in FORVAD** |
|  |  |

| **Summary of interview content** | **Notes/ideas/themes** |
| --- | --- |
| Views on/expectations of the trial |  |
| Experience of set-up |  |
| Patient recruitment (facilitators/barriers) |  |
| Patient pathway/integrating with standard practice |  |
| Delivering treatments |  |
| Follow up/outcomes |  |
| Views on brachialgia/surgical approach |  |
| Views on trials in brachialgia/neurosurgery |  |
| Other |  |
