# Supplementary material for: Rapid qualitative analysis of recruitment obstacles in the FORVAD (Posterior Cervical Foraminotomy surgery versus Anterior Cervical Discectomy surgery in the treatment of cervical brachialgia) randomised, controlled trial
Source: Trials. 2024 Aug 17;25:546. doi: 10.1186/s13063-024-08391-4 (PMC11330054; doi:10.1186/s13063-024-08391-4)
Supplement: Supplementary file 3 — Additional file 3 [file 13063_2024_8391_MOESM3_ESM.docx]

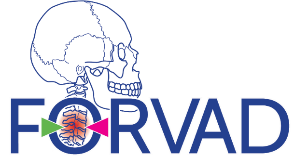


**Normalisation Process theory coding framework used for qualitative analysis of surgical RCT experiences for Healthcare Professionals**

**Healthcare Professionals**

| **Coherence** | **Cognitive Participation** | **Collective Action** | **Reflexive Monitoring** |
| --- | --- | --- | --- |
| **Differentiation**: Is there a clear understanding of how ACD differs from PCF in practice? And trial from off-trial pathways? | **Enrolment**: Are HCP’s interested in the research question enough to want to take part? | **Skill set workability**: How are tasks delegated to HCP’s during the trial and is this appropriate to the skill set of the clinical staff? | **Reconfiguration:** Do the results of the trial alter clinical equipoise regarding surgery enough to change practice? |
| **Communal specification**: do HCP’s have sufficient information – e.g., Aims, objectives, benefits of trial to develop shared understanding of the trial during recruitment? | **Activation**: Who is involved and to what degree throughout the trial in order to make it a success? | **Contextual Integration:** Is there enough support within the site to allow for effective set up and recruitment? (e.g., how PI engages others in the trial). | **Communal appraisal:** How do HCP’s perceive the value of the trial with regards to learning more about ACD and PCF? |
| **Individual specification**: Do HCP’s have sufficient understanding of their role within the trial and how will they go about this? | **Initiation**: How willing are individuals to invest in the trial and what are the barriers/facilitators to effect trial set up? | **Interactional workability:**  How is the trial operationalised within the daily clinical practice setting? | **Individual appraisal:**  How do HCP’s appraise the effects on them and their work environment when taking part in a surgical trial? |
| **Internalization**: Do HCP’s understand the importance of the trial and how this can contribute towards future research and development in neurosurgery? | **Legitimisation**: Is there sufficient equipoise amongst clinical staff for each site to be involved? | **Relational Integration:** Do individuals have confidence in the type of surgery performed? | **Systemisation:** How will learning more about the FORVAD trial be for learning more about surgical trials in general? |
