# Supplementary material for: Rapid qualitative analysis of recruitment obstacles in the FORVAD (Posterior Cervical Foraminotomy surgery versus Anterior Cervical Discectomy surgery in the treatment of cervical brachialgia) randomised, controlled trial
Source: Trials. 2024 Aug 17;25:546. doi: 10.1186/s13063-024-08391-4 (PMC11330054; doi:10.1186/s13063-024-08391-4)
Supplement: Supplementary file 4 — Additional file 4 [file 13063_2024_8391_MOESM4_ESM.pdf]

North West - Greater Manchester Central Research Ethics Committee
3rd Floor
Barlow House
4 Minshull Street
Manchester
M1 3DZ

Please note: This is the favourable opinion of the REC only and does not allow the amendment to be implemented at NHS sites in England until the outcome of the HRA assessment has been confirmed.

10 February 2021

Miss Fiona Brudenell Straw
Leeds Institute of Clinical Trials, Clinical Trials Research Unit
University of Leeds
Leeds
LS 2 9JT

Dear Miss Brudenell Straw

**Study title:**	**Clinical and Cost-effectiveness of Posterior Cervical Foraminotomy versus Anterior Cervical Discectomy in the Treatment of Cervical Brachialgia: A Multicentre, Phase III, Randomised Controlled Trial (FORVAD Trial)**
**REC reference:**	**18/NW/0682**
**Protocol number:**	**1.0**
**Amendment number:**	**SA01**
**Amendment date:**	**19/01/2021**
**IRAS project ID:**	**249138**

The above amendment was reviewed by the Sub-Committee in correspondence.

Ethical opinion

The members of the Committee taking part in the review gave a favourable ethical opinion of the amendment on the basis described in the notice of amendment form and supporting documentation.

No ethical issues were raised.

Approved documents

The documents reviewed and approved at the meeting were:

Document	*Version*	*Date*
Completed Amendment Tool [FORVAD Amendment Tool_Substantial Amendment 1]	V1.0	19 January 2021
Covering letter on headed paper [FORVAD REC Cover Letter_Substantial Amendment 1]	V1.0	29 January 2021
Interview schedules or topic guides for participants [FORVAD Interview Sub-study_Staff Interview Topic Guide]	V1.0	15 January 2021
Interview schedules or topic guides for participants [FORVAD Interview Sub-study_Patient Interview Topic Guide]	V1.0	15 January 2021
Letters of invitation to participant [FORVAD Interview Sub-study_Patient Cover Letter]	V1.0	15 January 2021
Letters of invitation to participant [FORVAD Interview Sub-study_Patient Reminder Cover Letter]	V1.0	15 January 2021
Participant consent form [FORVAD Interview Sub-study_Patient Telephone Interview Consent Checklist and Script]	V1.0	15 January 2021
Participant consent form [FORVAD Interview Sub-study_Staff Telephone Interview Consent Checklist and Script]	V1.0	15 January 2021
Participant information sheet (PIS) [FORVAD Interview Sub-study_Participant Information Sheet_Staff]	V1.0	15 January 2021
Participant information sheet (PIS) [FORVAD Interview Sub-study_Participant Information Sheet_Expression of Interest_For Patients]	V1.0	15 January 2021
Research protocol or project proposal [FORVAD Protocol (clean version)]	V3.0	15 January 2021
Research protocol or project proposal [FORVAD Protocol (tracked changes)]	V3.0	15 January 2021

Membership of the Committee

The members of the Committee who took part in the review are listed on the attached sheet.

Working with NHS Care Organisations

Sponsors should ensure that they notify the R&D office for the relevant NHS care organisation of this amendment in line with the terms detailed in the categorisation email issued by the lead nation for the study.

Amendments related to COVID-19

We will update your research summary for the above study on the research summaries section of our website. During this public health emergency, it is vital that everyone can promptly identify all relevant research related to COVID-19 that is taking place globally. If you have not already done so, please register your study on a public registry as soon as possible and provide the HRA with the registration detail, which will be posted alongside other information relating to your project.

Statement of compliance

The Committee is constituted in accordance with the Governance Arrangements for Research Ethics Committees and complies fully with the Standard Operating Procedures for Research Ethics Committees in the UK.

HRA Learning

We are pleased to welcome researchers and research staff to our HRA Learning Events and online learning opportunities– see details at: https://www.hra.nhs.uk/planning-and-improving-research/learning/

**IRAS Project ID - 249138:**	**Please quote this number on all correspondence**

Yours sincerely

pp 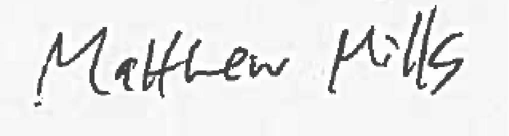

Dr George Gkimpas
Chair

E-mail: gmcentral.rec@hra.nhs.uk

Enclosures:	*List of names and professions of members who took part in the review*
Copy to:	*Mr Simon Thomson, Leeds Teaching Hospitals NHS Trust*

North West - Greater Manchester Central Research Ethics Committee

Attendance at Sub-Committee of the REC meeting on 05 February 2021

Committee Members:

Name	*Profession*	*Present*	*Notes*
Dr George Gkimpas	Clinical Fellow	Yes	(Chaired the meeting)
Miss Claire Williams	Head of Pharmacovigilance and Regulatory Services	Yes	

Also in attendance:

Name	*Position (or reason for attending)*
Mr Matthew Mills	Approvals Administrator
Miss Chelsea Philips	Approvals Administrator
